# Higher Daily Physical Activities Continue to Preserve Muscle Strength After Mid-Life, But Not Muscle Mass After Age of 75

**DOI:** 10.1097/MD.0000000000003809

**Published:** 2016-06-03

**Authors:** An-chun Hwang, Yu-Rui Zhan, Wei-Ju Lee, Li-Ning Peng, Liang-Yu Chen, Ming-Hsien Lin, Li-Kuo Liu, Liang-Kung Chen

**Affiliations:** From the Center for Geriatrics and Gerontology (A-cH, Y-RZ, L-NP, L-YC, M-HL, L-KL, L-KC), Taipei Veterans General Hospital; Department of Rehabilitation Medicine (Y-RZ), Taipei City Hospital Hoping Fuyou Branch; Department of Family Medicine (W-JL), Taipei Veterans General Hospital Yuanshan Branch; Aging and Health Research Center (A-cH, W-JL, L-NP, L-YC, M-HL, L-KL, L-KC); and Institute of Public Health (A-cH, W-JL, L-NP, L-YC, L-KC), National Yang Ming University, Taipei, Taiwan.

## Abstract

The objective of this study is to explore the impact of aging and daily physical activities (PA) on muscle mass and muscle strength among community-dwelling people in Taiwan.

The design is a cross-sectional study. Setting is a population-based community study.

One thousand eight hundred thirty-nine community-dwelling people aged 50 years and older in Taiwan participated in the study.

Measurements include demographic characteristics, Charlson Comorbidity Index (CCI) for multimorbidity, mini-nutritional assessment (MNA) for nutritional evaluation, functional autonomy measurement system (SMAF) for functional capacity, Chinese version mini mental state examination (MMSE), 5-item Taiwan Geriatric Depression Scale (TGDS-5), Chinese version of International Physical Activity Questionnaire (IPAQ), height-adjusted skeletal muscle index (SMI) by dual-energy X-ray absorptiometry, handgrip strength, timed 6-m walking test for usual gait speed. Laboratory measurements include testosterone, sex-hormone binding globulin (SHBG), dehydroepiandrosterone sulfate (DHEA-S), insulin-like growth factor-1 (IGF-1), high-sensitivity C-reactive protein (hsCRP), 25-OH vitamin D, and insulin resistance.

After adjusted for age, the lowest PA tertile was associated with multimorbidity, poorer functional capacity and nutritional status, more depressive symptoms, lower SMI and lower handgrip strength, and lower free androgen index (FAI) in men. The negative association between PA and low SMI was more significant among subjects aged younger than 65 and the association decreased with older age. For subjects aged younger than 65, moderate daily PA (Q2) group had lower risk of low SMI compared with Q1 participants (OR: 0.62, 95% CI = 0.39–0.98, *P* = 0.040). For muscle strength, higher daily PA was associated with lower risk of low handgrip strength after age of 65 and the effect was dose-dependent. The effect was attenuated by potential confounders during age 65 to 74, while after age 75, the result was almost unchanged in fully adjusted model (OR = 0.37, 95% CI = 0.18–0.79, *P* = 0.010).

Older age may attenuate the protective effects of higher daily PA on preventing muscle loss, but higher daily PA continues to preserve muscle strength at different age groups, even after the age of 75. The prognostic role of daily PA may be mediated by muscle strength instead of muscle mass among people aged 75 years and older.

## INTRODUCTION

Along with aging, the loss of skeletal muscle mass was present after the 5th decade of life with an average annual rate of 1% to 2%, which would accelerate after the 6th decade of life and even faster after age of 75.^1,2^ From age 50 to 80, the average number of muscle fiber and motor unit in the vastus lateralis decreased for more than 50% of that of younger men.^3^ Since skeletal muscle mass played an important role in muscle performance, the muscle mass decline was accompanied by at least an equal, but usually greater, loss in muscle strength.^1,4,5^

Sarcopenia has been described as the age-related decline in skeletal muscle mass plus low muscle strength and/or low physical performance.^6^ Sarcopenia has been reported to be associated with higher risk of falls,^7^ functional decline or disability.^8–10^ mortality,^11,12^ and poorer outcome among hospitalized older adults.^13,14^ The development of sarcopenia was a multifactorial process, which may be greatly accelerated by physical inactivity because physical inactivity was a mediator for both loss of muscle mass and muscle strength.^15^

The effect of exercise intervention or daily physical activity (PA) on muscle mass and strength was generally considered positive.^16,17^ Nevertheless, whether the benefits may offset the age-related decline was less investigated. Younger population who were more physically active had higher muscle mass, and lower body fat,^18^ and frail older adults with structured exercise intervention still had significant gains in muscle size and strength.^19,20^ If the effect of exercise intervention could be interpreted as that of higher daily PA was much more elusive. It has been reported the association between PA and muscle strength was stronger in “younger” old people rather than the “older” old people and in “women” than “men.”^21,22^

There were several other contributing factors for low muscle mass or strength in addition to older age and physical inactivity during the process of aging, such as multimorbidities,^23^ cognitive function,^24^ nutritional status,^25^ hormones,^26,27^ inflammation and wasting,^28^ and insulin resistance.^29^ Many of the abovementioned factors may confound the relationship between daily PA, muscle mass and function, especially in the observational study. Besides, aging is a highly heterogenous process and the “young old” may be regarded as physiologically different population from the “old old.”

Therefore, the main aim of this study was to investigate the complex interrelationship between aging and daily PA on muscle mass and muscle strength among community-dwelling people across 3 age strata (50–64, 65–74, over 75) after adjustment for various potential confounders that may be overlooked in previous studies.

## METHODS

### Study Population

The present study used the first-wave sampling data of I-Lan Longitudinal Aging Study (ILAS), collected from Aug, 2011 to Aug, 2013 for cross-sectional analysis. ILAS was designed to elucidate the interrelationship between aging, frailty, sarcopenia, and cognitive decline in Taiwan.^30^

Residents aged over 50 in the Yuanshan Township of I-Lan County were stratified by every 5 years of age with 25% sampling rate and 70% response rate. They were enrolled when they fully consented and agreed to participate. The survey included 1839 community-dwelling older people for final analysis.

Subjects with the following conditions were excluded for participation: unable to communicate with the research staff to complete the interview, functionally dependent, that is, unable to finish 6-m walk within a reasonable period of time, limited life expectancy (<6 months) due to major illnesses, currently institutionalized, or contraindicated for brain magnetic resonance imaging plan to move out of the communities in the near future. The whole study had been approved by the Institutional Review Board of National Yang Ming University.

### Anthropometry and Demographic Measurements

A questionnaire encompassing information about demographic characteristics including educational level, smoking and alcohol consumption, and medical history was completed by well-trained research staff. Disease was defined by self-report plus use of related medications. Charlson Comorbidity Index (CCI) was used for estimation of multimorbidity. Besides, functional capacity, cognitive function, nutritional status, and depressive symptoms were evaluated by functional autonomy measurement system (SMAF), mini-mental state examination (MMSE), mini-nutrition assessment (MNA), and 5-item Taiwan Geriatric Depression Scale (TGDS-5), respectively.

### Physical Activity, Skeletal Muscle Mass, and Muscle Strength

In this study, Taiwanese version of International Physical Activity Questionnaire (IPAQ) was used to quantify PA.^31^ IPAQ scores of men and women were categorized into sex-specific tertiles in the entire studied sample. Handgrip strength for each subject was measured using digital dynamometers (Smedlay's Dynamo Meter; TTM, Tokyo, Japan) in an upright standing position, with arms down by their sides. The best result of 3 tests of dominant hand was recorded as the handgrip strength (HS). All participants received the whole body dual-energy X-ray absorptiometry scan to measure lean body mass by a Lunar Prodigy instrument (GE Healthcare, Madison, WI). Appendicular skeletal muscle mass (ASM) was defined as the sum of the lean body mass of 4 limbs. The skeletal muscle index (SMI) was expressed as ASM divided by height square as recommended by previous studies.^32^ The lowest quintile of SMI and handgrip strength by sex were defined as low skeletal muscle mass, and low muscle strength.

### Laboratory Measurements

All study participants received 20-cc whole blood sampling after overnight fast. Insulin-like growth factor-1 (IGF-1, ng/mL) and total testosterone (ng/dL) were analyzed by chemiluminescence immunoassay analyzer (DPC Immulite 2000, Siemens, USA and ADVIA Centaur, Siemens, USA respectively). SHBG and dehydroepiandrosterone sulfate (DHEA-S, ug/dL) were measured by electrochemiluminometry (Roche Elecsys e411; Roche, Indianapolis, IN). Free androgen index (FAI) was defined as total testosterone (nM/L) divided by sex hormone binding globulin (SHBG, nM/L).

High-sensitivity C-reactive protein (hsCRP, mg/dL) was measured by an automatic analyzer (ADVIA 1800, Siemens, USA). Serum level of insulin (uIU/mL) and 25-OH vitamin D(25-OH VitD, ng/mL) were analyzed by chemiluminescence immunoassay (ADVIA Centaur, Siemens, USA, and Diasorin Liaison, Italy respectively). Insulin resistance was estimated by the Homeostatic model assessment model (HOMA-IR) and was calculated as (glucose (mg/dL) × insulin (uIU/mL))/405.

### Statistical Analysis

For main variables including SMI, handgrip strength, and PA, data of handgrip strength and PA were available among all 1839 participants; while 1779 of them had measurements of SMI. In this study, comparisons of continuous variables between groups were done by analysis of covariance (ANCOVA) with adjustment of age, while chi-square test was applied to compare categorical variables. After testing the interaction between PA and age on low SMI and low handgrip strength, the statistical interaction was significant in both models (*P* value both <0.001). Thus, we stratified the participants into 3 age groups (50–64 years old, 65–74 years old, over 75 years old). Binary logistic regression was used to evaluate the association between PA with low skeletal muscle mass, and low muscle strength across different age strata. Model 1 was adjusted for age and sex; models 2 and 3 were further adjusted for significant variables of laboratory measurement and functional plus comorbidity assessment individually with *P* value less than 0.1 shown in Table 1. Since skeletal muscle mass played an important role in grip strength, model 4 included all of the adjustments of model 3 plus low SMI. All statistical analyses were performed by commercial statistical software (SPSS 20.0, Chicago, IL). A *P* value less than 0.05 (2-tailed) was considered statistically significant.

**TABLE 1:**
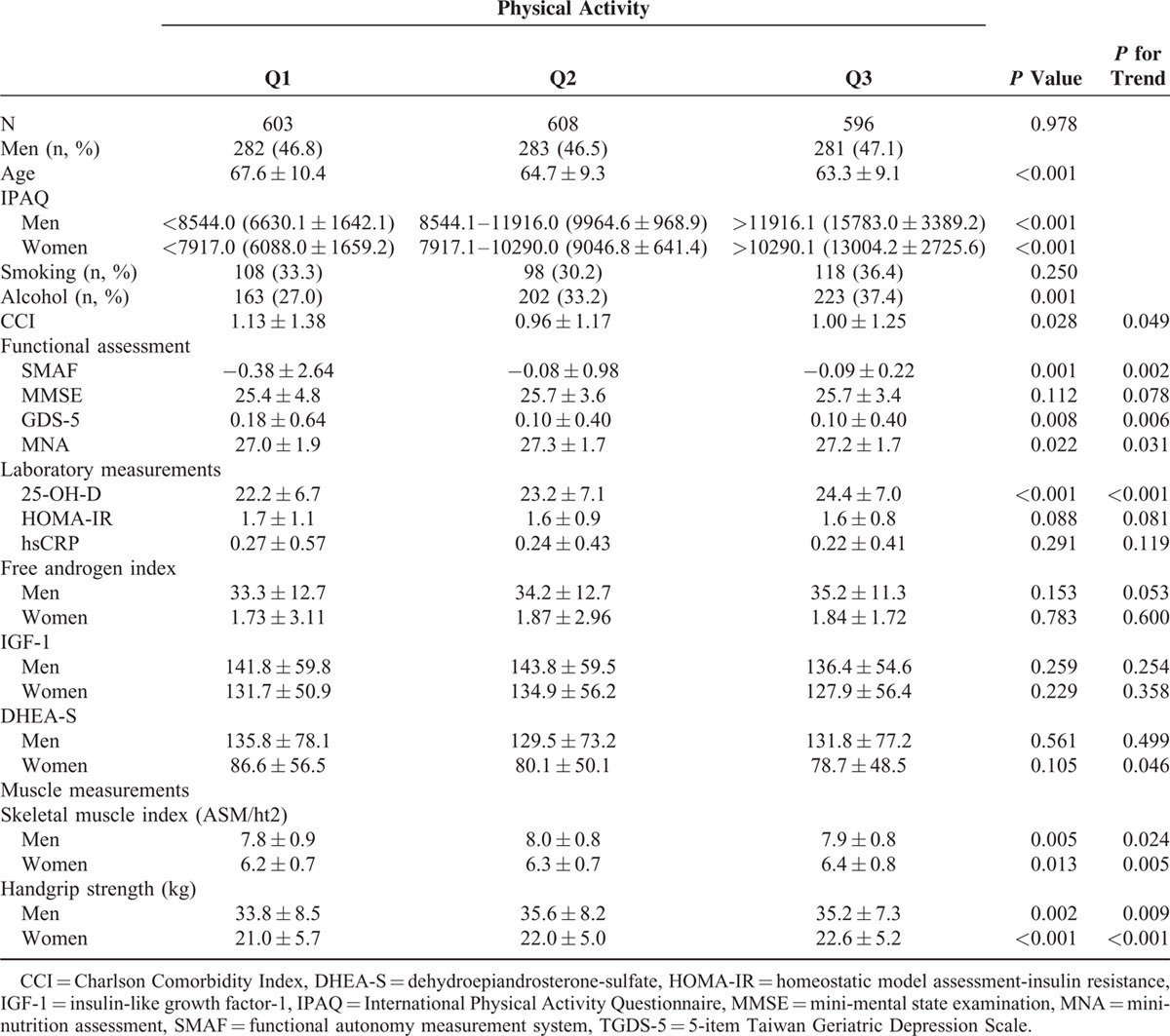
Baseline Characteristics of Study Participants, Stratified by Physical Activity

## RESULTS

Overall, data of 1839 participants (mean age: 63.9 ± 9.3 years, 47.5% male) were retrieved for analysis. Table 1 summarizes the baseline characteristics of the participants stratified by their PA tertiles. After adjustment for age, lower tertile of PA was associated with higher CCI (*P* for trend = 0.049), poorer functional capacity (*P* for trend = 0.002), more depressive symptoms (*P* for trend = 0.006), and lower MNA scores (*P* for trend = 0.031). Besides, participants with low PA also had lower serum level of 25-OH-VitD (*P* for trend <0.001), lower FAI in men and higher DHEA-S in women individually (*P* for trend = 0.053 for FAI in men, and 0.046 for DHEA-S in women). In both sexs, skeletal muscle index (SMI) and handgrip strength also increased with PA (SMI: *P* for trend = 0.024 in men and 0.005 in women; handgrip strength: *P* for trend = 0.009 in men and <0.001 in women). In addition, participants with higher levels of PA were more likely to be current alcohol drinker (*P* = 0.001).

Table 2 demonstrates the associations between PA and low SMI across different age groups. Generally, the inverse association of PA and low SMI was more significant among subjects aged younger than 65 and the effect decreased with older age. For subjects aged younger than 65, moderate PA(Q2) group had lower risk of low SMI compared with their Q1 counterparts (crude OR = 0.55, 95% CI = 0.36–0.84, *P* = 0.005), and the result was consistent across adjusted models (OR = 0.62, 95% CI = 0.39–0.98, *P* = 0.04 in model 3). Nevertheless, after adjustment for potential confounding factors, age *per se* was not associated with low SMI among 3 age groups.

**TABLE 2:**
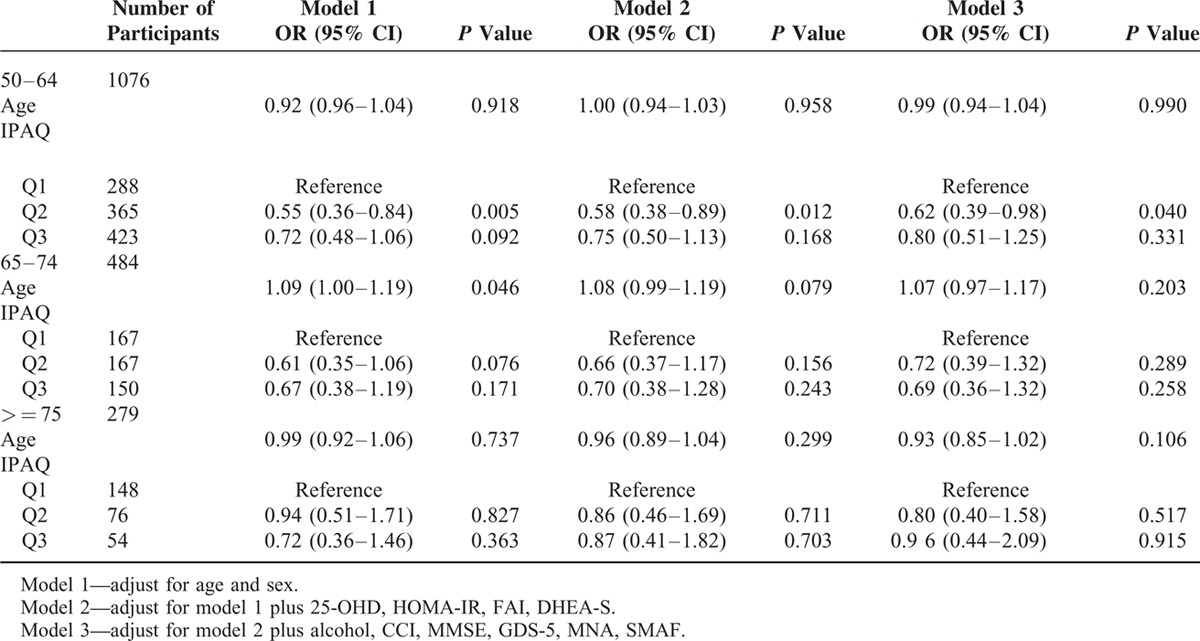
Multiple Logistic Regression on Low Skeletal Muscle Mass, Stratified by Age Group

Table 3 demonstrates the associations between PA and low handgrip strength across age strata. Higher PA was significantly correlated with lower risk of low handgrip strength after 65 and the effect was dose-dependent. Compared with the participants with lowest tertile (Q1) of PA, the crude odds ratio (OR) of low handgrip strength of Q2 and Q3 was 0.58 (95% CI 0.36–0.94, *P* = 0.026) and 0.56 (95% CI 0.34–0.94, *P* = 0.026) among age 65–74; 0.72 (95% CI 0.40–1.29, *P* = 0.268) and 0.35 (95% CI 0.18–0.67, *P* = 0.002) among age over 75. The effect was attenuated by potential confounders during age 65 to 74 (OR = 0.71, 95% CI 0.42–1.20, *P* = 0.205 in Q2; OR = 0.65, 95% CI 0.37–1.14, *P* = 0.130 in Q3) in model 4. After age of 75, the association of highest level of PA and low handgrip strength remained unchanged in fully adjusted model (OR = 0.37, 95% CI = 0.18–0.79, *P* = 0.010 in model 4).

**TABLE 3:**
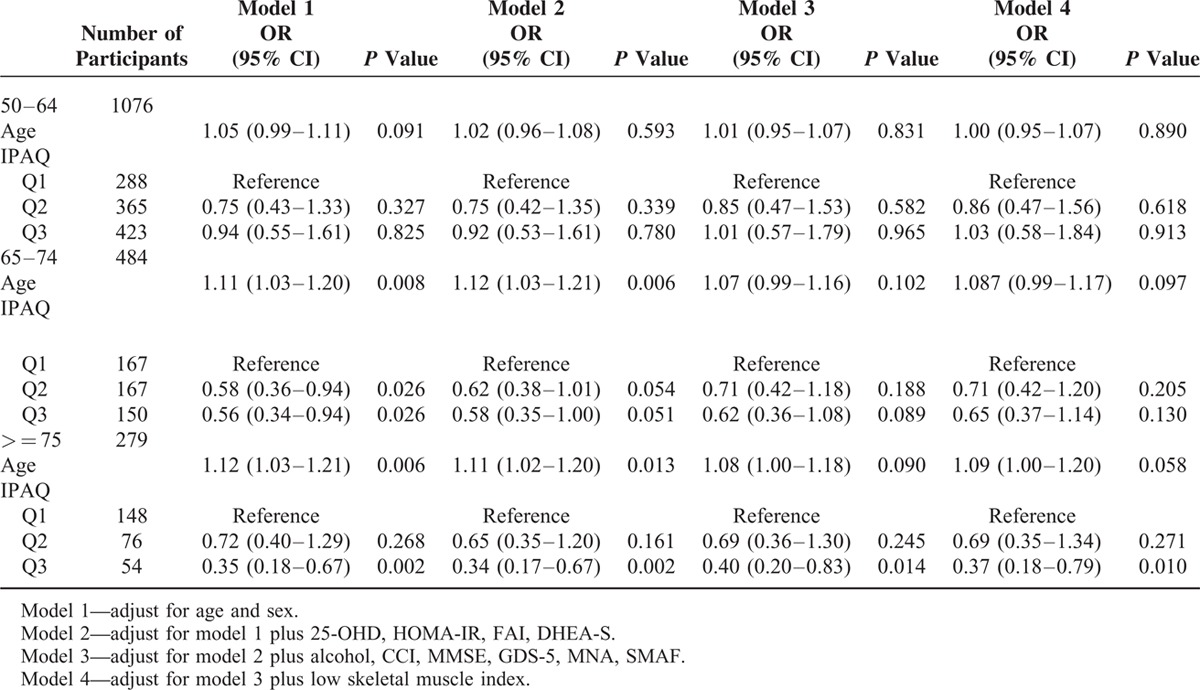
Logistic Regression on Low Skeletal Muscle Strength, Stratified by Age Group

The significant contributing factors for low SMI or low handgrip strength in model 3 are summarized in Table 4. Poorer nutritional status was significantly associated with higher risk of low SMI and low handgrip strength across age groups. Along with the increase in MNA score, the OR of low SMI was 0.61 (95% CI 0.54–0.68, *P* <0.001), 0.63 (95% CI 0.53–0.74, *P* <0.001), and 0.78 (95% CI 0.67–0.91, *P* = 0.001) in ages 50–64, 65–74, and over 75 respectively; while for low handgrip strength, the OR was 0.86 (95% CI 0.76–0.98, *P* = 0.026), 0.85 (95% CI 0.75–0.97, *P* = 0.019), and 0.89 (95% CI 0.77–1.03, *P* = 0.111) for 3 age groups. The negative association between serum 25-OH-VitD levels and low SMI increased with aging (OR = 0.97, 95% CI 0.94–1.01, *P* = 0.158 in age 64–75; OR = 0.95, 95% CI 0.91–1.00, *P* = 0.035 in age over 75). After age 65, lower MMSE score was positively associated with lower handgrip strength (OR = 0.89, 95% CI 0.84–0.95, *P* <0.001 in age 64–75; OR = 0.93, 95% CI 0.87–1.00, *P* = 0.004 in age over 75). Among measured hormone profiles, free androgen index was inversely associated with low SMI before age 75 (OR = 0.96, 95% CI 0.93–0.99, *P* = 0.004 in age 50–64; OR = 0.97, 95% CI 0.94–1.00, *P* = 0.068 in age 65–74), and low handgrip strength before age 65 (OR = 0.95, 95% CI 0.91–0.99, *P* = 0.008). DHEA-S was negatively correlated with low SMI and handgrip strength primarily after 75 (OR = 0.99, 95% CI 0.99–1.00, *P* = 0.039 for low SMI; OR = 0.99, 95% CI 0.99–1.00, *P* = 0.099 for low handgrip strength).

**TABLE 4:**
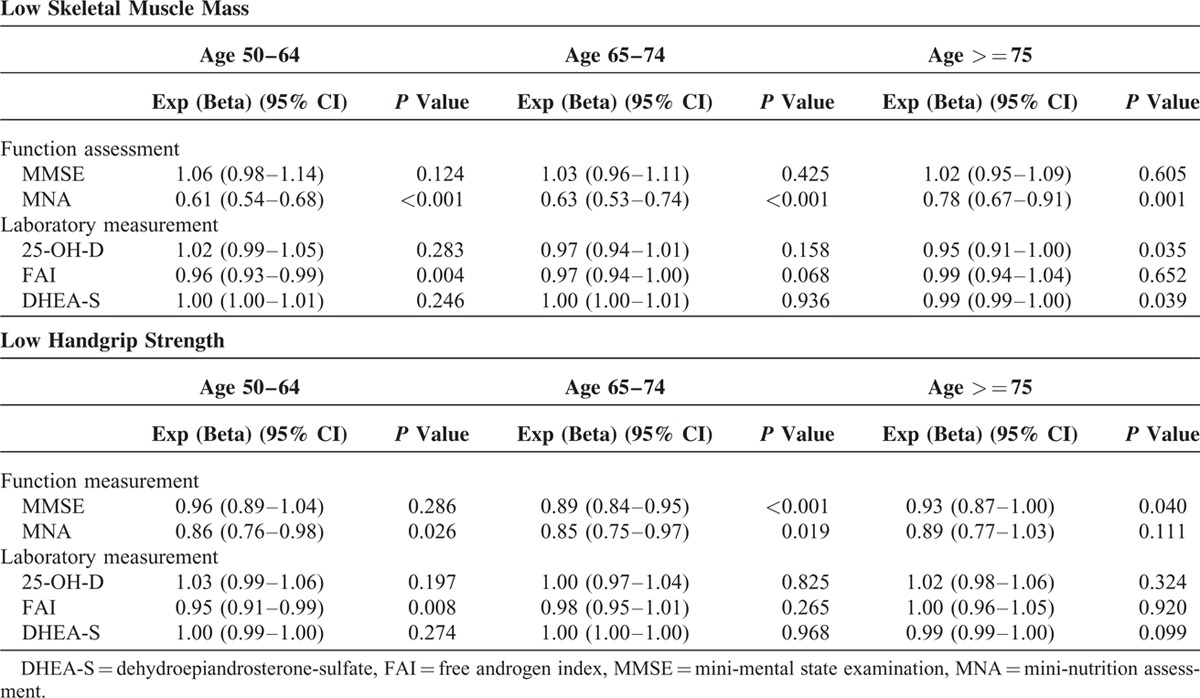
Independent Factors for Low Skeletal Muscle Mass or Low Handgrip Strength

## DISCUSSION

### Physical Activity and Muscle Mass

The impact of higher daily PA on increasing skeletal muscle mass has been reported in younger adults,^18^ however, whether the effect could be extended to older age remained to be controversial. It has been proposed that maintaining regular PA may not counteract the age-related loss of appendicular skeletal muscle mass in the older population.^33,34^ In this study, the inverse association of high daily PA on low skeletal muscle mass was significant before age 65, and then declined along with aging, especially after 75. For the nonlinear relationship between PA and low SMI before age 65, we do additional analysis with adjustment of smoking status. The prevalence of smoking was the highest among the most physically active (Q3) group, which may attenuate the protective effect of high physical activity. The odds ratio of Q2 and Q3 of model 1 was 0.57 (95% CI 0.38–0.84, *P* value = 0.005) and 0.67 (95% CI 0.46–0.99, *P* value = 0.042) respectively. The result of model 2 and 3 did not change significantly, possibly due to inadequate statistical power. Smoking and other unmeasured confounders may explain the nonlinear relationship between physical activity and risk of low skeletal muscle in the age group of 50 to 64. Published recent study from Japan supported regular walking with ankle weight (0.5 kg) improved skeletal muscle mass in old adults with mean age of 75.^35^ Short et al^36^ also reported structured aerobic exercise training could enhance muscle protein synthesis regardless of age. The above findings implied that maintaining skeletal muscle mass in advanced age required extra aerobic or resistance exercise training in addition to daily PA. In this study, the impact of age on low SMI in each age group attenuated significantly in fully adjusted model, which suggested that loss of skeletal muscle mass may be slowed down by correcting modifiable risk factors.

### Physical Activity and Muscle Strength

On the contrary, the advantage of high PA on preserving skeletal muscle strength was very significant after age of 65 and the effect was dose-dependent. Although the previous study reported that the association was stronger in younger old adults,^22^ results of this study showed the consistent correlation regardless of older age, even more significant after age 75 for the participants with highest PA tertile. It echoed the work from Fiatarone et al,^37^ which reported that the resistance training could improve muscle strength and functional mobility among nursing home-living nonagenarians. The effect of high PA on muscle strength was gradually attenuated after adjustment for functional and hormonal measurement and low SMI among participants aged 65 to 74. Nevertheless, the effect remained almost unchanged over age 75, which was of great importance to functional capacity. In addition to quantity of SMI, activation of neuromuscular junction^38^ and mitochondrial function^39^ were potential mediators, which may partially explain the independent effect of PA on muscle strength.

### Hormone and Muscle Mass or Strength

A great body of literature have supported the effect of hormones on skeletal muscle mass and strength.^27,40,41^ Our previous study has shown that free androgen index was increased along with muscle mass and muscle strength among both sexs aged 50 years and older.^42^ In this study, the negative association of FAI and low skeletal muscle mass was primarily seen before age of 75, while DHEA-S took the place after that. For low handgrip strength, the impact of FAI was more significant for subjects aged younger than 65. Overall, the importance of androgen on muscle strength decreased during aging after adjustment for PA.

### Nutrition and Muscle Mass or Strength

The impact of nutrition on muscle health during aging has been investigated extensively in recent years, especially the role of adequate protein intake.^43^ Malnutrition significantly increased the risk of low SMI and low handgrip strength after age of 50 in this study. The association between malnutrition and low handgrip strength attenuated significantly after adjustment for low SMI. The odds ratio was 0.90 (95% CI 0.79–1.04, *P* = 0.154), 0.87 (95% C.I 0.76–1.01, *P* = 0.061), 0.95(95% C.I 0.81–1.11, *P* = 0.498) among people aged 50–64, 65–74, over 75 individually (data not shown). This suggested that adequate nutritional intake may preserve muscle quality (strength) by means of increment of quantity (muscle mass). The association between 25(OH)D and muscle health in older people has been inconclusive, while recent reviews supported the beneficial effects of muscle strength by vitamin D supplement for those with serum 25(OH)D less than 25 nmol/L.^44–46^ In this study, the association between 25-OH-D and skeletal muscle was limited to mass instead of strength when PA was taken into account, and the effect was even more significant in advanced age.

### Limitations

Despite all the efforts that went into the study, there were several limitations. First, the study was currently a cross-sectional design. Since the follow-up data were not available yet, the causal relationship between aging, PA, skeletal muscle mass, and muscle strength was unable to be confirmed. Longitudinal follow-up data would help to clarify the impact of PA on skeletal muscle mass loss. Second, lack of objective measures on the level and intensity of PA may underestimate the association between active lifestyle and the outcomes. However, this is a common condition in the previous literature. Third, it was less clear whether the threshold of daily PA to improve muscle function exists. Since most study participants were living a physically active lifestyle, so the benefits of daily PA may be underestimated.

## CONCLUSION

The protective effect of high daily PA on age-related skeletal muscle mass decline diminished along with aging. Without structured exercise straining, maintaining active lifestyle per se was associated with lower risk of muscle weakness, which was of great importance to maintain functional independence and most important of all, the effect persisted after age of 75. Further study is needed to evaluate the prognostic role of high daily PA in advanced age, as well as the mediating roles of muscle mass and muscle strength.
